# Siegesbeckiae Herba Extract and Chlorogenic Acid Ameliorate the Death of HaCaT Keratinocytes Exposed to Airborne Particulate Matter by Mitigating Oxidative Stress

**DOI:** 10.3390/antiox10111762

**Published:** 2021-11-04

**Authors:** Jae Won Ha, Yong Chool Boo

**Affiliations:** Department of Molecular Medicine, School of Medicine, BK21 Plus KNU Biomedical Convergence Program, Cell and Matrix Research Institute, Kyungpook National University, 680 Gukchaebosang-ro, Jung-gu, Daegu 41944, Korea; jaewon1226@knu.ac.kr

**Keywords:** Siegesbeckiae Herba, *Siegesbeckia pubescens* Makino, airborne particulate matter, PM_10_, glutamate-cysteine ligase, nuclear factor erythroid 2-related factor 2, glutathione, chlorogenic acid, caffeic acid

## Abstract

Airborne particulate matter with a size of 10 μm or less (PM_10_) can cause oxidative damages and inflammatory reactions in the skin. This study was conducted to discover natural products that are potentially useful in protecting the skin from PM_10_. Among the hot water extracts of a total of 23 medicinal plants, Siegesbeckiae Herba extract (SHE), which showed the strongest protective effect against PM_10_ cytotoxicity, was selected, and its mechanism of action and active constituents were explored. SHE ameliorated PM_10_-induced cell death, lactate dehydrogenase (LDH) release, lipid peroxidation, and reactive oxygen species (ROS) production in HaCaT cells. SHE decreased the expression of KEAP1, a negative regulator of NRF2, and increased the expression of NRF2 target genes, such as HMOX1 and NQO1. SHE selectively induced the enzymes involved in the synthesis of GSH (GCL-c and GCL-m), the regeneration of GSH (GSR and G6PDH), and GSH conjugation of xenobiotics (GSTκ1), rather than the enzymes that directly scavenge ROS (SOD1, CAT, and GPX1). SHE increased the cellular content of GSH and mitigated the oxidation of GSH to GSSG caused by PM_10_ exposure. Of the solvent fractions of SHE, the n-butyl alcohol (BA) fraction ameliorated cell death in both the absence and presence of PM_10_. The BA fraction contained a high amount of chlorogenic acid. Chlorogenic acid reduced PM_10_-induced cell death, LDH release, and ROS production. This study suggests that SHE protects cells from PM_10_ toxicity by increasing the cellular antioxidant capacity and that chlorogenic acid may be an active phytochemical of SHE.

## 1. Introduction

Air pollution, exacerbated by rapid climate change and industrial development, poses a major environmental factor that threatens human health [1]. Particulate matter (PM) suspended in the atmosphere is a complex material that contains various organic compounds and heavy metals [2]. Continued exposure to high concentrations of PM increases the incidence of various diseases, including respiratory and cardiovascular diseases, and mortality [3]. PM with an approximate diameter of less than 10 or 2.5 μm is called PM_10_ and PM_2.5_, respectively [4].

As the interface between the body and the environment, the skin acts as a barrier to protect our body from environmental pollutants, but it is an organ directly exposed to environmental pollutants. The pores of the skin are large enough for PM to penetrate [5], and children with immature skin or patients with compromised skin barriers are relatively vulnerable to the percutaneous absorption and damage of PM compared to healthy adults [6,7,8]. PM exacerbates inflammatory skin diseases, such as atopic dermatitis, acne, and psoriasis by various mechanisms [3,9].

PM exposure stimulates Ca^2+^ signaling and increases the production of reactive oxygen species (ROS), such as superoxide radical (O_2_^•−^), hydrogen peroxide (H_2_O_2_), and hydroxyl radical (^•^OH) [10]. Aryl hydrocarbons contained in PM increase the production of ROS in the process of metabolism [11]. Transition metal components also catalyze chemical reactions that generate ROS [12]. PM stimulates the NADPH oxidase family to increase ROS production [13]. Among the NADPH oxidase family, dual oxidase 2 has been reported to mediate the generation of ROS stimulated by PM_10_ or house dust mites [14,15].

PM enhances the expression of cyclooxygenase 2 and increases the production of the eicosanoid mediator prostaglandin E_2_ [16,17]. In addition, it stimulates the cell signaling system to increase the secretion of inflammatory cytokines, such as tumor necrosis factor-α, interleukin (IL)-1β, IL-6, and IL-8 [18]. PM also stimulates the expression of matrix metalloproteinases (MMPs), leading to increased degradation of the extracellular matrix, such as collagen [19]. Thus, there is a need for antioxidative countermeasures against skin inflammation and aging due to PM.

Plants are a good source of natural products with biological activities that are potentially useful for maintaining skin health and beauty [20,21,22,23]. It should be noted that certain phytochemicals can act as either antioxidants or prooxidants and have a positive or negative effect on cell survival depending on their type and concentration [24,25,26,27]. Phytochemicals that can reduce cellular oxidative stress directly or indirectly are expected to provide a useful strategy to reduce PM-induced skin inflammation and aging [28]. They can directly scavenge ROS or enhance cellular antioxidant capacity by inducing nuclear factor erythroid 2-related factor (NRF) 2-mediated gene expression [29]. In a previous study, our research team found that several phenolic compounds derived from terrestrial and marine plants, such as punicalagin, (−)-epigallocatechin-3-gallate, and dieckol relieve oxidative damage and reduce ROS production in HaCaT cells stimulated by PM_10_ and inhibit the subsequent cell signaling process and inflammatory responses [17,18,26].

This study aimed to discover a natural product that effectively relieves PM_10_-induced cytotoxicity and oxidative stress. To this end, the effects of several medicinal plant extracts on the viability of HaCaT keratinocytes exposed to PM_10_ were compared. As a result, Siegesbeckiae Herba extract (SHE) among the extracts of 23 medicinal plants, most effectively defended against PM_10_ cytotoxicity. Siegesbeckiae Herba generally refers to the dried aerial parts of *Siegesbeckia orientalis* L, *Siegesbeckia pubescens* Makino, and *Siegesbeckia glabrescens* Makino, and has been used as a traditional medicine in Korea, Japan, China, and Vietnam [30]. Studies on the phytochemicals and biological efficacy of SHE have been increasing recently [31,32]. However, studies on the protective action of SHE against PM cytotoxicity have not yet been reported. In the present study, the antioxidant properties of SHE prepared from the dried leaves of *Siegesbeckia pubescens* Makino were investigated in HaCaT cells exposed to PM_10_, with a special focus on the mechanism of action and active constituents. The results of this study suggest that SHE increases cell defense gene expression and thereby mitigates cell death and oxidative damage caused by PM_10_. It was also suggested that chlorogenic acid rather than caffeic acid may be the active constituent providing the cell protection effect.

## 2. Materials and Methods

### 2.1. Reagents

Standardized fine dust (PM_10_-like) (European Reference Material ERM-CZ120), chlorogenic acid (Cat. C3878), and caffeic acid (Cat. C0625) were purchased from Sigma-Aldrich (St. Louis, MO, USA).

### 2.2. Extracts of Medicinal Plants

Medicinal plant extracts were obtained from the Plant Extract Bank of Korea (https://portal.kribb.re.kr/kpeb) (Cheongju, Korea). The plant sources and catalog numbers of the extracts used in this study are as follow; Castaneae Semen, CW01-011; Eucomiae Folium, CW01-052; Peucedani Japonici Radix, CW01-065; Melonis Calyx, CW02-003; Arisaematis Rhizoma, CW02-006; Eucomiae Ramulus, CW02-011; Dioscoreae Rhizoma, CW02-051; Mori Ramulus, CW02-060; Pruni Humilidis Semen, CW02-085; Angelicae tenuissimae Radix, CW03-008; Fagopyri Semen, CW03-011; Aconiti Jaluencis Tuber, CW03-082; Biotae Orientalis Folium, CW03-084; Gardeniae Fructus, CW03-087; Vitis Viniferae Caulis, CW03-100; Benincasae Semen, CW04-004; Akebiae Caulis, CW04-006; Pini Ramulus, CW04-021; Pini Pollen, CW04-022; Machili Thunbergi Cortex, CW04-056; Cyperi Rhizoma, CW04-069; Glycine Semen nigra, CW04-098; Siegesbeckiae Herba, CW04-100.

### 2.3. Siegesbeckiae Herba Extract and Its Solvent Fractions

Dried Siegesbeckiae Herba (*Siegesbeckia pubescens* Makino) was purchased from Sinsun Herb (http://sinsunherb.co.kr) (Seoul, Korea) and its extract was prepared in this laboratory. Dried leaves (140 g) were ground and extracted with 0.9 L water at 90 °C for 1 h. The extracted solution was evaporated under reduced pressure to obtain the crude extract (9 g). The SHE was dispersed in 150 mL water and partitioned sequentially with an equal volume of methylene chloride (MC), ethyl acetate (EA), and n-butyl alcohol (BA). Evaporation of the organic solvents yielded MC fraction (0.27 g), EA fraction (0.16 g), and BA fraction (0.46 g). The aqueous layer was filtered to remove insoluble material (1.15 g) and then evaporated to obtain a water (WT) fraction (6.64 g).

### 2.4. High-Performance Liquid Chromatography with Photodiode Array Detection (HPLC-DAD)

HPLC-DAD analysis was carried out using a Waters Alliance HPLC system (Waters, Milford, MA, USA) consisting of an e2695 separation module and a 2996 photodiode array detector. The stationary phase was a Hector-M C_18_ column (4.6 mm × 250 mm, 5 m) (RS Tech Co., Daejeon, Korea). The mobile phase was a mixture of 0.1% phosphoric acid (A) and acetonitrile (B) with the following composition: 0–30 min, a linear gradient from 0 to 100% B; 30–40 min, 100% B. The solvent gradient program was as follows: 0–30 min, a linear gradient from 0 to 100% B; 30–40 min, 100% B; 40–45 min, a linear gradient from 100 to 0% B. The flow rate of the mobile phase was 0.6 mL min^−1^. The sample injection volume was 10 μL.

### 2.5. Cell Culture and PM_10_ Treatment

HaCaT cells, an immortalized human keratinocyte cell line originally established by Dr. Fusenig [33], were obtained from Dr. In-San Kim (Kyungpook National University, Daegu, Korea) and cultured in a closed incubator at 37 °C in humidified air containing 5% CO_2_. Cells were administered DMEM/F-12 medium (GIBCO-BRL, Grand Island, NY, USA) containing 10% fetal bovine serum, 100 U mL^−1^ penicillin, 100 μg mL^−1^ streptomycin, 0.25 μg mL^−1^ amphotericin B, and 10 μg mL^−1^ hydrocortisone every three days. Cells were cultured on 96-well, 12-well, or 6-well culture plates (SPL Life Sciences, Pocheon, Korea) for at least 24 h and then treated with either test materials, PM_10_, or both, at the specified concentrations for up to 48 h.

### 2.6. Cell Viability and Lactate Dehydrogenase (LDH) Release Assays

Cell viability was assessed by a method using 3-(4,5-dimethylthiazol-2-yl)-2,5-diphenyl tetrazolium bromide (MTT) [34]. Cells were plated onto 96-well culture plates at 4 × 10^3^ cells/well and maintained in a 200 μL culture medium for 24 h. The cells were treated with a vehicle or a test material and cultured in the absence or presence of PM_10_ (200 μg mL^−1^) for 48 h. After discarding or saving the conditioned medium, adherent cells were incubated in a 100 μL growth medium containing 1 mg mL^−1^ MTT (Amresco, Solon, OH, USA) for 2 h in an incubator. After removing the medium and washing the cells with phosphate-buffered saline (PBS), the dye was extracted from the cells with 100 μL dimethyl sulfoxide, and the absorbance of the extracts was measured at 570 nm with a SPECTROstar nano microplate reader (BMG LABTECH GmbH, Ortenberg, Germany).

The saved medium was used in the assay for LDH release using the TaKaRa LDH cytotoxicity detection kit (TaKaRa Bio Inc., Shiga, Japan). Briefly, 50 µL aliquot of the medium diluted 2-times with PBS was mixed with 50 µL of the reaction mixture prepared according to manufacturer protocol (this mixture contains sodium lactate, diaphorase, NAD^+^, and iodotetrazolium chloride). The final reaction mixture was incubated for 30 min at 25 °C and the absorbance was measured at 490 nm with a SPECTROstar nano microplate reader.

### 2.7. Cellular Lipid Peroxidation Assay

Cellular lipid peroxidation was assessed using a 2-thiobarbituric acid (TBA) method [35]. The cells were plated onto 6-well culture plates at 2 × 10^5^ cells/well and maintained in a 2 mL culture medium for 24 h. The cells were treated with vehicle or test material and cultured in the absence or presence of PM_10_ (200 μg mL^−1^) for 48 h. After discarding the medium and washing the cells with PBS, the adherent cells were lysed using the lysis buffer A (20 mM Tris-Cl, 2.5 mM ethylenediamine-*N*,*N*,*N*′,*N*′-tetraacetic acid, 1.0% sodium dodecyl sulfate, pH 7.5). The assay mixture consisted of 100 μL cell lysate, 50 μL 1.0% *meta*-phosphoric acid, and 350 μL 0.9% TBA (Sigma-Aldrich), which was then heated in a boiling water bath for 45 min. After cooling, 500 μL *N*-butyl alcohol (BA) was added to the mixture, which was then vortex-mixed and centrifuged at 13,000 rpm for 15 min to produce two separate layers. The fluorescence intensity of the BA layer (excitation at 544 nm and emission at 590 nm) was measured by using a Gemini EM fluorescence microplate reader (Molecular Devices, Sunnyvale, CA, USA). Data are presented as thiobarbituric acid-reactive substance (TBARS) levels corrected for protein contents. A standard curve was prepared using 1,1,3,3-tetramethoxypropane (Sigma-Aldrich) as a donor of malondialdehyde.

### 2.8. Cellular ROS Production Assay

Cellular ROS production was assessed by using 2′,7′-dichlorodihydrofluorescein diacetate (DCFH-DA), a cell-permeable fluorescent dye sensitive to changes in the redox state of a cell [36]. The cells were plated onto 12-well culture plates at 8 × 10^4^ cells/well for 24 h. Cells were pre-labeled with 10 μM DCFH-DA (Sigma-Aldrich) for 60 min and treated with 200 μg mL^−1^ PM_10_ alone or in combination with a test material at different concentrations for 60 min. Cells were washed twice with PBS and the images of cells fluorescing due to the oxidation of DCFH-DA were obtained with a LEICA DMI3000 B microscope (Leica Microsystems GmbH, Wetzlar, Germany). The dye was extracted from cells using the lysis buffer A (150 μL/well). The extracted solution was centrifuged at 13,000 rpm for 15 min and the supernatant was used for the measurement of fluorescence intensity (excitation at 485 nm and emission at 538 nm) with a Gemini EM fluorescence microplate reader.

### 2.9. Glutathione (GSH) and Glutathione Disulfide (GSSG) Assay

GSH and GSSG contents were measured in a recycling assay using 5,5′-dithio-bis-2-nitrobenzoic acid (DTNB) [37]. Cells were plated onto 6-well culture plates at 2 × 10^5^ cells/well and maintained in a 2 mL culture medium for 24 h. The cells were treated with vehicle or test material and cultured in the absence or presence of PM_10_ (200 μg mL^−1^) for 24 h. Cells were extracted using 5% meta-phosphoric acid (150 μL per well). The extracted solution was centrifuged at 13,000 rpm for 15 min and the supernatant was used for the measurement of GSH/GSSG, using a GSH/GSSG assay kit (product number GT40) from Oxford Biomedical Research (Oxford, UK). Total GSH plus GSSG content was measured using the extract as it is, and the GSSG content was quantified after pre-scavenging GSH in the extract with a pyridine derivative. Absorbance change due to reduction of DTNB was measured at 412 nm, and a calibration curve was prepared using a GSSG standard. The GSH content was calculated by subtracting the GSSG content from the total GSH plus GSSG content.

### 2.10. Quantitative Reverse Transcriptase-Polymerase Chain Reaction (qRT-PCR)

The mRNA levels of catalase (CAT), glucose 6-phosphate dehydrogenase (G6PDH), glutamate-cysteine ligase catalytic subunits (GCL-c), glutamate-cysteine ligase modifier subunit (GCL-m), glutathione disulfide reductase (GSR), glutathione peroxidase (GPX) 1, glutathione S-transferase (GST) κ1, heme oxygenase (HMOX) 1, kelch-like ECH-associated protein (KEAP) 1, NRF2, NAD(P)H quinone oxidoreductase (NQO) 1, and superoxide dismutase (SOD) 1 and were determined by qRT-PCR using a StepOnePlus Real-Time PCR System (Applied Biosystems, Foster City, CA, USA). The cells were plated onto 6-well culture plates at 2 × 10^5^ cells/well and maintained in a 2 mL culture medium for 24 h. The cells were treated with vehicle or test material and cultured in the absence or presence of PM_10_ (200 μg mL^−1^) for 24 h. Total cellular RNA was extracted from cells with an RNeasy kit (Qiagen, Valencia, CA, USA), and this RNA was used as a template for the synthesis of complementary DNA with a high-capacity cDNA archive kit (Applied Biosystems). Gene-specific primers for qRT-PCR were purchased from Macrogen (Seoul, Korea), and their nucleotide sequences are shown in Table 1. The qRT-PCR reaction mixture (20 µL) consisted of SYBR Green PCR Master Mix (Applied Biosystems), complementary DNA (60 ng), and gene-specific primer sets (2 picomole). Thermal cycling parameters were set as follows: 50 °C for 2 min, 95 °C for 10 min, 40 amplification cycles of 95 °C for 15 s and 60 °C for 1 min, and a dissociation step. In each run, the melting curve analysis confirmed the homogeneity of the PCR product. The mRNA levels of each gene were calculated relative to that of the internal reference, glyceraldehyde 3-phosphate dehydrogenase (GAPDH), using the comparative Ct method [37]. Ct is defined as the number of cycles required for the PCR signal to exceed the threshold level. Fold changes in the test group compared to the control group were calculated as 2^−∆∆^^Ct^, where ∆∆Ct = ∆Ct_(test)_ − ∆Ct_(control)_ = (Ct_(gene, test)_ − Ct_(reference, test)_) − (Ct_(gene, control)_ − Ct_(reference, control)_).

### 2.11. Western Blotting

Western blotting was performed as previously described [47]. Primary antibodies for GCL-c (#390811), GCL-m (#55586), G6PDH (#373886), and β-actin (#47778) were purchased from Santa Cruz Biotechnology (Santa Cruz, CA, USA). Anti-rabbit IgG (#2357) and anti-goat IgG (#2020) secondary antibodies were purchased from Santa Cruz Biotechnology, and anti-mouse IgG (#7076) secondary antibody was purchased from Cell Signaling Technology (Danvers, MA, USA). Antibodies were diluted in TBST (137 mM sodium chloride, 20 mM Tris, 0.1% Tween 20, pH 7.6.) containing 5% skim milk. Proteins in cell lysate samples were denatured by adding Laemmli 5× sample buffer and heating at 95 °C for 5 min. Proteins (40 μg) were resolved with 10% SDS-polyacrylamide gel electrophoresis at 80 V and electrically transferred to a polyvinylidene difluoride membrane (Amersham Pharmacia, Little Chalfont, UK) at 4 °C overnight. After blocking incubation with TBST containing 5% skim milk, the membrane was incubated with the primary antibody at 4 °C overnight, followed by incubation with the secondary antibody at room temperature for 1 h. The target protein bands were visualized with a chemiluminescence method using the picoEPD Western Reagent kit (ELPIS-Biotech, Daejeon, Korea). The captured blot images were analyzed using the Image J program from U.S. National Institutes of Health (Bethesda, MD, USA).

### 2.12. Statistical Analysis

Data are expressed as mean ± standard deviation (SD) of three or more independent experiments. Experimental results were statistically analyzed using SigmaStat v.3.11 software (Systat Software Inc., San Jose, CA, USA). The presence of significantly different group means was determined using a one-way analysis of variance (ANOVA) at *p* < 0.05 level. Dunnett’s test was then used to compare each experimental group with the control group. Alternatively, Duncan’s multiple range test was used to compare all groups to each other.

## 3. Results

### 3.1. Effects of Medicinal Plant Extracts on the PM_10_-Induced Toxicity in HaCaT Keratinocytes

**Figure 1:**
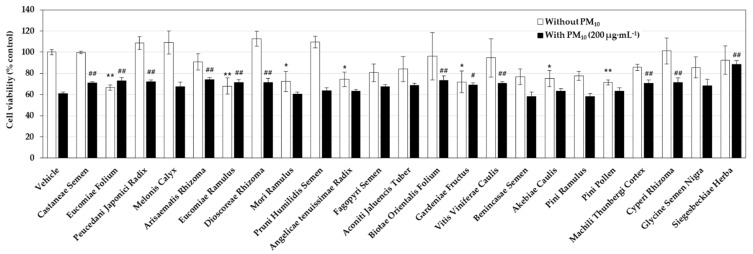
Effects of medicinal plant extracts on the viability of HaCaT keratinocytes cultured in the absence and presence of particulate matter with an approximate diameter of less than 10 μm (PM_10_). Cells were pretreated with vehicle or each extract (50 μg mL^−1^) and cultured in the absence or presence of PM_10_ (200 μg mL^−1^) for 48 h. Cell viability was determined by the 3-(4,5-dimethylthiazol-2-yl)-2,5-diphenyl tetrazolium bromide (MTT) assay. Data are presented as mean ± SD (*n* = 3). Statistical significance of intergroup differences was determined using one-way ANOVA followed by Dunnett’s test. * *p* < 0.05 and ** *p* < 0.01 versus vehicle control; # *p* < 0.05 and ## *p* < 0.01 versus PM_10_ only control.

A preliminary experiment was performed to select plant extracts that alleviate the cytotoxicity of PM_10_. HaCaT cells were treated with each plant extract at 50 μg mL^−1^ and exposed to 200 μg mL^−1^ PM_10_ for 48 h. As shown in Figure 1, in the case of the vehicle control group, the cell viability decreased by about 40% by PM_10_ exposure. Of the 23 plant extracts tested, seven plant extracts showed their own toxicity, significantly reducing cell viability in the absence of PM_10_; they are the extracts derived from Eucomiae Folium, Eucomiae Ramulus, Mori Ramulus, Angelicae tenuissimae Radix, Gardeniae Fructus, Akebiae Caulis, and Pini Pollen. On the other hand, the nine plant extracts had no effect on cell viability by themselves, and also mitigated the decrease in cell viability caused by PM_10_; they are the extracts derived from Castaneae Semen, Peucedani Japonici Radix, Arisaematis Rhizoma, Dioscoreae Rhizoma, Biotae Orientalis Folium, Vitis Viniferae Caulis, Machili Thunbergi Cortex, Cyperi Rhizoma, and Siegesbeckiae Herba. Among them, Siegesbeckiae Herba extract (SHE) most effectively restored the viability of PM_10_-exposed cells, so this extract was selected for subsequent experiments.

### 3.2. Effects of SHE on the Viability, LDH Release, Lipid Peroxidation, and ROS Production in HaCaT Cells Exposed to PM_10_

**Figure 2:**
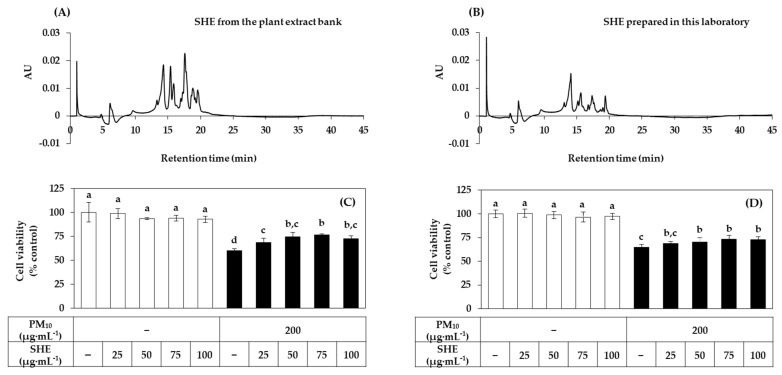
Comparison of Siegesbeckiae Herba extracts (SHEs) purchased from the plant extract bank and prepared in this laboratory. For the comparison of phytochemical composition, high-performance liquid chromatography (HPLC) profiles of the SHEs from the plant extract bank (**A**) and prepared in this laboratory (**B**) are shown (concentration 0.1%). For the comparison of bioactivity, HaCaT keratinocytes were treated with SHEs from the plant extract bank (**C**) and prepared in this laboratory (**D**) at different concentrations and cultured in the absence or presence of PM_10_ (200 μg mL^−1^) for 48 h. Cell viability was determined by the MTT assay. Data are presented as mean ± SD (*n* = 4). Duncan’s multiple range test was performed to compare all group means to each other. Groups that share the same letters (a–d) do not have significantly different means at the *p* < 0.05 level.

SHE used in the preliminary experiment was purchased from an external plant extract bank. We also manufactured SHE from the dried leaves of *Siegesbeckia pubescens* Makino in our laboratory. Figure 2A,B shows the HPLC patterns of SHE purchased from an external plant extract bank and SHE prepared in our laboratory. The two extracts appeared to share several peaks with the same retention times, while the heights of these peaks were somewhat different. The effects of the two extracts on the viability of HaCaT cells in the presence or absence of PM_10_ exposure were compared in Figure 2C,D. Both extracts did not affect cell viability by themselves at the concentrations tested, and significantly increased the viability of cells exposed to PM_10_ to some degree. These results indicate that the phytochemical composition and biological activity of the two extracts are similar to each other. In subsequent experiments, SHE manufactured in our laboratory was used.

**Figure 3:**
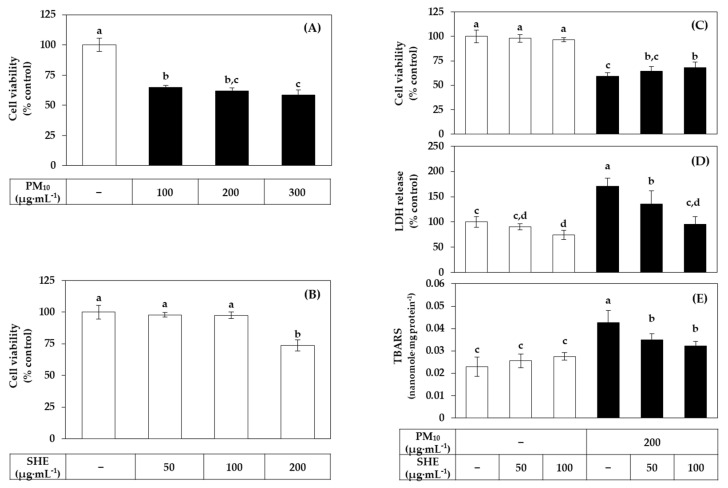
Effects of SHE on the viability, lactate dehydrogenase (LDH) release, and lipid peroxidation in HaCaT keratinocytes exposed to PM_10_. In (**A**,**B**), cells were treated with PM_10_ (**A**) or SHE (**B**) at different concentrations for 48 h. In (**C**–**E**), cells were treated with SHE at different concentrations and cultured in the absence or presence of PM_10_ (200 μg mL^−1^) for 48 h. Cell viability was determined by the MTT assay (**A**–**C**). LDH release was assessed using the conditioned medium (**D**). Lipid peroxidation of cell lysates was determined by the 2-thiobarbituric acid assay and data are presented as 2-thiobarbituric acid-reactive substance (TBARS) levels corrected for protein contents (**E**). Data are presented as mean ± SD (*n* = 5 for (**A**–**D**); *n* = 4 for (**E**)). Duncan’s multiple range test was performed to compare all group means to each other. Groups that share the same letters (a–d) do not have significantly different means at the *p* < 0.05 level.

Figure 3A,B shows the dose curves of PM_10_ and SHE on HaCaT cell viability. PM_10_ showed a gradual increase in toxicity up to 300 μg mL^−1^. In this study, PM_10_ was treated at 200 μg mL^−1^ as this concentration induced consistent and substantial cytotoxicity. SHE had no effect on the cell viability up to 100 μg mL^−1^ but reduced it at 200 μg·mL^−1^. We selected the PM_10_ treatment concentration (200 μg·mL^−1^) that showed sufficient toxicity for the experimental purpose, and the SHE treatment concentrations (50 μg mL^−1^ and 100 μg mL^−1^) were selected in the concentration range that did not reduce the cell viability. In Figure 3C–E, SHE treatment at 50 μg mL^−1^ and 100 μg mL^−1^ inhibited the decrease in cell viability, the increase in LDH release, and the increase in lipid peroxidation induced by PM_10_ treatment at 200 μg mL^−1^.

**Figure 4:**
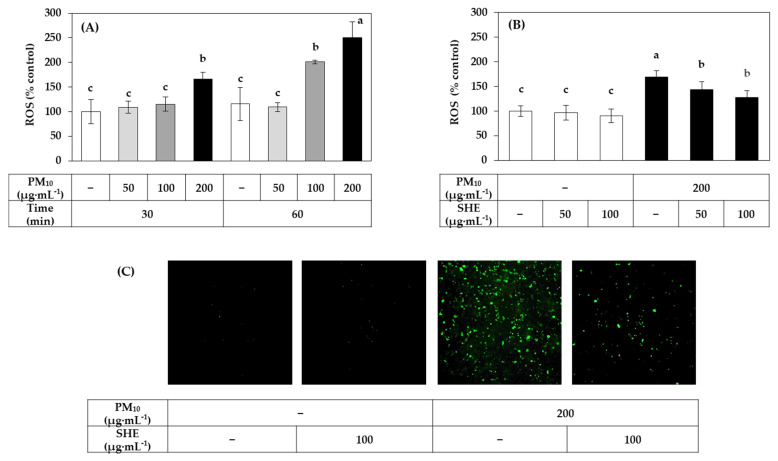
Effects of SHE on the reactive oxygen species (ROS) production in HaCaT keratinocytes exposed to PM_10_. In (**A)**, cells were labeled with 2′,7′-dichlorofluorescin diacetate (DCFH-DA) and exposed to PM_10_ at the indicated concentrations for 30 min or 60 min. In (**B**,**C**), cells were labeled with DCFH-DA, treated with SHE at the indicated concentrations, and exposed to PM_10_ (200 μg mL^−^^1^) for 60 min or not. Typical images of cells fluorescing due to the oxidation of DCFH-DA by ROS are shown in (**C**). The fluorescence of the cell extracts was measured to determine ROS levels (**A**,**B**). Data are presented as mean ± SD (*n* = 5 for (**A**) and *n* = 6 for (**B**)). Duncan’s multiple range test was performed to compare all group means to each other. Groups that share the same letters (a–c) do not have significantly different means at the *p* < 0.05 level.

Figure 4A shows the PM_10_ dose and time-dependency of ROS production in HaCaT cells. In the following experiments, PM_10_ was treated at 200 μg mL^−1^ for 60 min as this condition induced ROS production at a substantial level. ROS production was detected using DCFH-DA, a redox-sensitive dye that fluoresces after being oxidized by ROS. As shown in Figure 4B, SHE treatment at 50 μg mL^−1^ and 100 μg mL^−1^ inhibited the increase in ROS production induced by PM_10_ treatment at 200 μg mL^−1^ for 60 min. Typical fluorescence pictures of cells treated differently are shown in Figure 4C. The fluorescence due to the oxidation of DCFH-DA was increased by PM_10_ and the change was reduced by SHE.

### 3.3. Effects of SHE on the Expression of the Defense Genes in HaCaT Cells under Basal and PM_10_-Exposed Conditions

**Figure 5:**
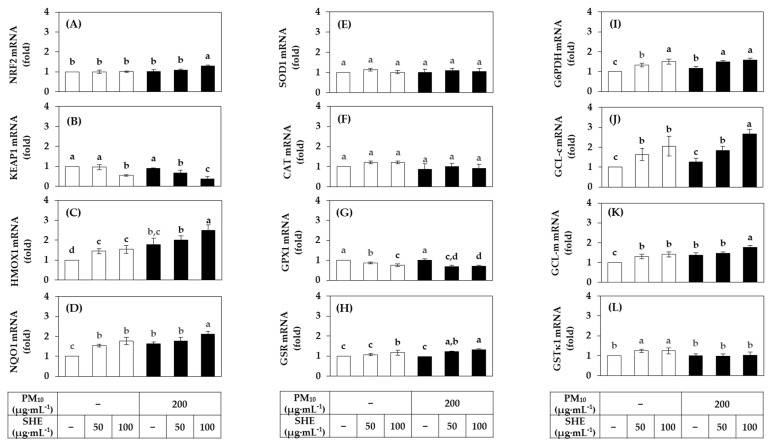
Effects of SHE on the mRNA expression levels of the defense genes in HaCaT keratinocytes under basal and PM_10_-exposed conditions. Cells were treated with SHE at different concentrations and cultured in the absence or presence of PM_10_ (200 μg mL^−1^) for 24 h. The mRNA levels of nuclear factor erythroid 2-related factor (NRF) 2 (**A**), kelch-like ECH-associated protein (KEAP) 1 (**B**), heme oxygenase (HMOX) 1 (**C**), NAD(P)H quinone oxidoreductase (NQO) 1 (**D**), superoxide dismutase (SOD) 1 (**E**), catalase (CAT) (**F**), glutathione peroxidase (GPX) 1 (**G**), glutathione disulfide reductase (GSR) (**H**), glucose 6-phosphate dehydrogenase (G6PDH) (**I**), glutamate-cysteine ligase catalytic subunit (GCL-c) (**J**), glutamate-cysteine ligase modifier subunit (GCL-m) (**K**), and glutathione S-transferase (GST) κ1 (**L**) were determined by quantitative real time-polymerase chain reaction (qRT-PCR) and normalized to that of glyceraldehyde 3-phosphate dehydrogenase (GAPDH). Data are presented as mean ± SD (*n* = 3). Duncan’s multiple range test was performed to compare all group means to each other. Groups that share the same letters (a, b, c, or d) do not have significantly different means at the *p* < 0.05 level.

To gain insight into the mechanism of action of SHE to alleviate cellular oxidative damage, we examined the effect of SHE on the mRNA expression of several defense enzymes in HaCaT cells in the absence or presence of PM_10_ exposure, and the results are shown in Figure 5. SHE did not have a remarkable effect on the expression of NRF2, a master transcription factor that induces the expression of various genes in the body’s antioxidant defense system, but decreased the expression of its negative regulator, KEAP1, both in the absence and presence of PM_10_ exposure. SHE further increased the mRNA expression of HMOX1 and NQO1, the main target genes of NRF2.

The mRNA expression of SOD1, which scavenges superoxide radicals, and CAT, which decomposes hydrogen peroxide, was not significantly affected by SHE. The mRNA expression of GPX1, which decomposes hydrogen peroxide or lipid peroxide, tended to be decreased by SHE, and that of GSTκ1, which catalyzes GSH conjugation of xenobiotics, showed a tendency to slightly increase. The mRNA expression of GSR, which catalyzes the reduction of GSSG coupled with NADPH oxidation, was increased by SHE, and the mRNA expression of G6PDH, which supplies NADPH, was also significantly increased. In addition, the mRNA expressions of GCL-m and GCL-c acting at the rate-regulating step of GSH biosynthesis were increased by SHE. These results suggest that SHE enhances cellular antioxidant capacity, including synthesis and regeneration of GSH.

**Figure 6:**
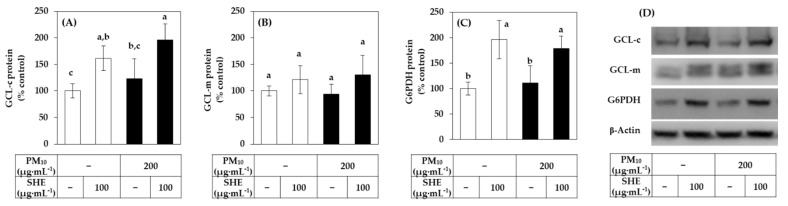
Effects of SHE on the protein levels of GCL-c, GCL-m, and G6PDH in HaCaT keratinocytes under basal and PM_10_-exposed conditions. Cells were treated with SHE (100 μg mL^−1^) and cultured in the absence or presence of PM_10_ (200 μg mL^−1^) for 24 h. The protein levels of GCL-c (**A**), GCL-m (**B**), and G6PDH (**C**) were determined by the Western blotting and normalized to that of β-actin. Representative blots are shown in (**D**). Data are presented as percentages of the control (mean ± SD, *n* = 3). Duncan’s multiple range test was performed to compare all group means to each other. Groups that share the same letters (a–c) do not have significantly different means at the *p* < 0.05 level.

Western blot was performed to analyze the protein level of GCL-c, GCL-m and G6PDH as the representative proteins, and β-actin as a reference protein. As shown in Figure 6, SHE increased the protein levels of GCL-c and G6PDH in both the absence and presence of PM_10_. It also tended to increase the protein level of GCL-m although the differences were not statistically significant. The changes in the protein levels of these proteins were consistent with those in the mRNA levels observed above.

### 3.4. Effects of SHE on the GSH and GSSG Levels in HaCaT Cells Exposed to PM_10_

**Figure 7:**
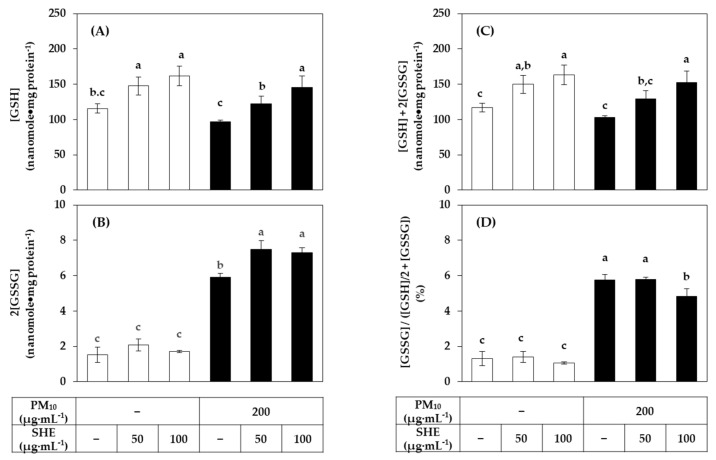
Effects of SHE on the contents of reduced glutathione (GSH) and its oxidized form, glutathione disulfide (GSSG) in HaCaT keratinocytes exposed to PM_10_. Cells were treated with SHE at different concentrations and cultured in the absence or presence of PM_10_ (200 μg mL^−1^) for 24 h. The GSH contents (**A**) were calculated by subtracting the GSSG contents (**B**) from the total GSH plus GSSG contents (**C**). The relative ratios of GSSG contents to the total GSH plus GSSG contents were presented in (**D**). Data are presented as mean ± SD (*n* = 3). Duncan’s multiple range test was performed to compare all group means to each other. Groups that share the same letters (a–c) do not have significantly different means at the *p* < 0.05 level.

The effect of SHE on the contents of GSH and its oxidized form, GSSG, in cells was investigated. As shown in Figure 7, SHE increased the GSH content in both the absence and presence of PM_10_. PM_10_ did not significantly affect the level of GSH, but significantly increased the level of GSSG. As a result, the total GSH plus GSSG content increased by SHE in a concentration-dependent manner. Interestingly, the proportion of GSSG in the total GSH and GSSG content was greatly increased by PM_10_ and this change was attenuated by SHE. This suggests that SHE enhances cell resistance to PM_10_-induced oxidative stress by increasing the synthesis of GSH in cells.

### 3.5. Effects of Solvent Fractions of SHE on the Viability of HaCaT Cells Exposed to PM_10_

**Figure 8:**
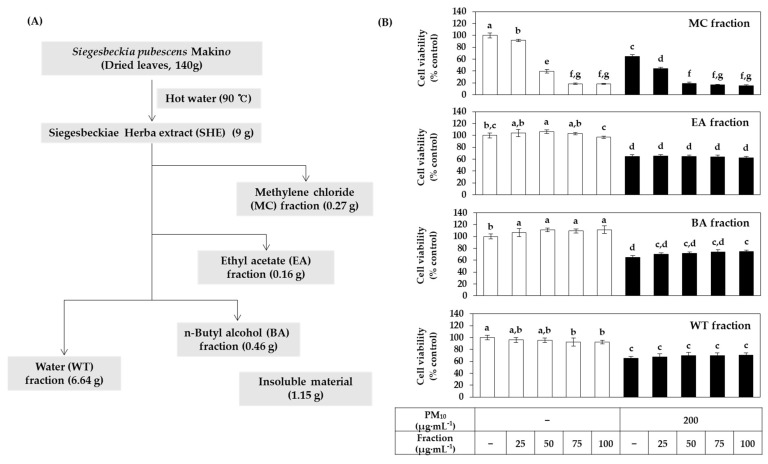
Effects of various solvent fractions of SHE on the viability in HaCaT keratinocytes exposed to PM_10_. (**A**) SHE was separated into methylene chloride (MC), ethyl acetate (EA), n-butyl alcohol (BA), and water (WT) fractions. (**B**) Cells were treated with each solvent fraction at different concentrations and cultured in the absence or presence of PM_10_ (200 μg mL^−1^) for 48 h. Cell viability was determined by the MTT assay. Data are presented as mean ± SD (*n* = 4). Duncan’s multiple range test was performed to compare all group means to each other. Groups that share the same letters (a–g) do not have significantly different means at the *p* < 0.05 level.

To investigate which phytochemicals contained in SHE alleviate the cytotoxicity of PM_10_, this extract was divided into several solvent fractions, namely, MC, EA, BA, and WT fractions according to the method illustrated in Figure 8A. Additionally, the effect of each fraction on the viability of HaCaT cells under PM_10_ exposure or non-exposure conditions was comparatively evaluated. As shown in Figure 8B, the MC fraction itself showed severe cytotoxicity. Although the EA fraction and WT fraction had weaker cytotoxicity, they did not alleviate PM_10_ cytotoxicity. Notably, the BA fraction increased the cell viability compared to the vehicle control in both the absence and presence of PM_10_.

### 3.6. HPLC-DAD Analysis of Solvent Fractions of SHE

**Figure 9:**
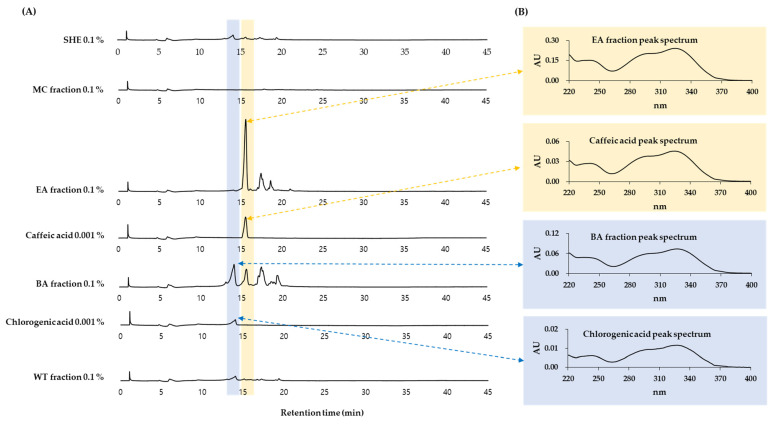
High-performance liquid chromatography-photodiode array detection (HPLC-DAD) analysis of SHE and its solvent fractions. SHE was separated into MC, EA, BA, and WT fractions. HPLC-DAD analysis conditions are described in Materials and Methods. Authentic chlorogenic acid and caffeic acid were used to identify the major peaks by comparing retention times and spectra. Chromatograms detected at 330 nm are shown in (**A**). UV absorption spectra of the indicated peaks are shown in (**B**).

HPLC-DAD analysis was performed on SHE and its MC, EA, BA, and WT fractions. As shown in Figure 9, it was shown that the BA fraction contained chlorogenic acid and the EA fraction contained caffeic acid. This was based on a comparison of retention times and absorption spectra of the specified peaks with standard materials.

### 3.7. Effects of Chlorogenic Acid vs. Caffeic Acid on the Viability, LDH Release, and ROS Production of HaCaT Cells Exposed to PM_10_

**Figure 10:**
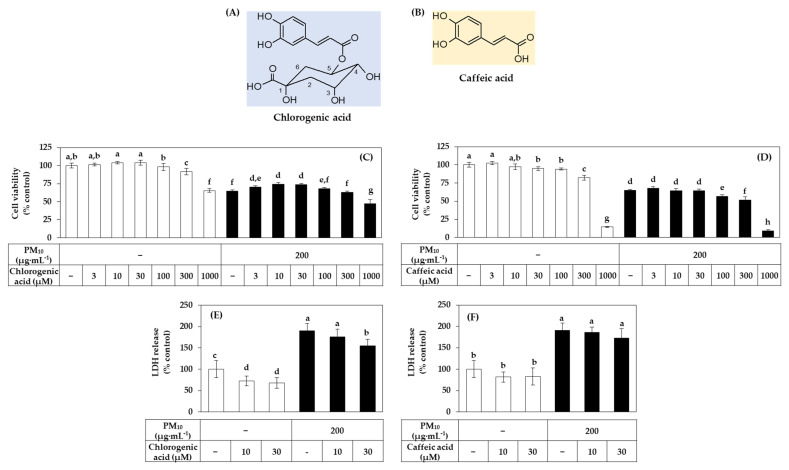
Effects of chlorogenic acid versus caffeic acid on the viability and LDH release of HaCaT keratinocytes exposed to PM_10_. The chemical structures of chlorogenic acid and caffeic acid are shown in (**A**,**B**). In (**C**–**F**), cells were treated with chlorogenic acid (**C**) or caffeic acid (**D**) at the specified concentrations and incubated in the absence or presence of PM_10_ (200 μg mL^−1^) for 48 h. Cell viability was determined by the MTT assay (**C**,**D**). LDH release was assessed using the conditioned medium (**E**,**F**). Data are presented as mean ± SD (*n* = 4 for (**C**,**D**); *n* = 5 for (**E**,**F**)). Duncan’s multiple range test was performed to compare all group means to each other. Groups that share the same letters (a–h) do not have significantly different means at the *p* < 0.05 level.

Chlorogenic acid is a compound of caffeic acid and quinic acid (Figure 10A,B). In the following experiments, we comparatively evaluated the effects of these two phytochemicals on the viability and ROS production of HaCaT cells under PM_10_ exposure or non-exposure conditions. As shown in Figure 10C, chlorogenic acid increased the cell viability compared to the vehicle control in both the presence and absence of PM_10_. However, this action was not observed with caffeic acid (Figure 10D).

The activity of inhibiting PM_10_-induced LDH release was found to be relatively superior with chlorogenic acid compared to caffeic acid (Figure 10E,F).

**Figure 11:**
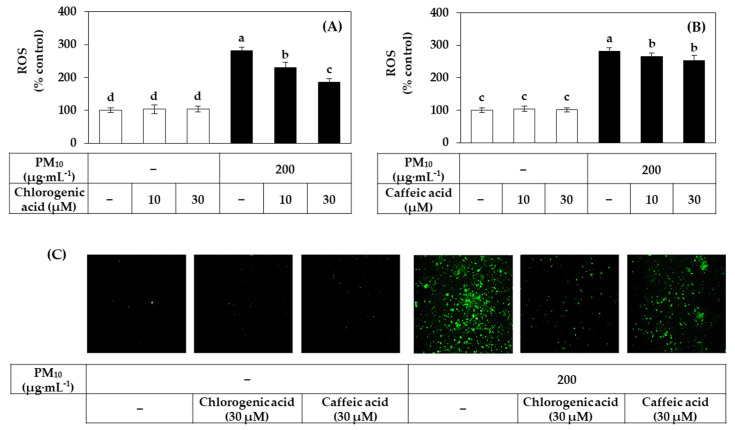
Effects of chlorogenic acid versus caffeic acid on the ROS production in HaCaT keratinocytes exposed to PM_10_. Cells were labeled with DCFH-DA, treated with SHE at different concentrations, and exposed to PM_10_ (200 μg mL^−1^) for 60 min or not. Typical images of cells fluorescing due to the oxidation of DCFH-DA by ROS are shown in (**C**). The fluorescence of the cell extracts was measured to determine ROS levels (**A**,**B**). Data are presented as mean ± SD (*n* = 4). Duncan’s multiple range test was performed to compare all group means to each other. Groups that share the same letters (a–d) do not have significantly different means at the *p* < 0.05 level.

Figure 11A,B shows the inhibitory effects of chlorogenic acid and caffeic acid on the ROS production induced by PM_10_. Among these two compounds, chlorogenic acid showed a stronger inhibitory effect against PM_10_-induced ROS production. Typical fluorescence pictures of cells are shown in Figure 11C. The fluorescence due to the oxidation of DCFH-DA by ROS was increased by PM_10_ and the change was reduced by chlorogenic acid more effectively than caffeic acid.

## 4. Discussion

**Figure 12:**
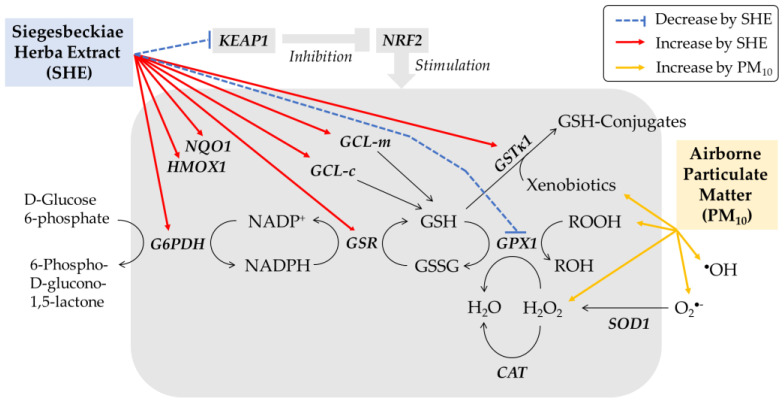
Working model for the protective effects of SHE against PM_10_-induced oxidative stress in HaCaT keratinocytes. PM_10_ increases ROS, such as superoxide radical (O_2_^•−^), hydrogen peroxide (H_2_O_2_), and hydroxyl radical (^•^OH), and lipid peroxide (ROOH), causing oxidative stress and death of HaCaT cells. SHE does not change the mRNA expression of NRF2, but decreases that of KEAP1, a negative regulator of NRF2, leading to the activation of NRF2 to induce the expression of target genes, such as HMOX1 and NQO1. SHE selectively induces the enzymes involved in the synthesis of GSH (GCL-c and GCL-m), the regeneration of GSH (GSR and G6PDH), and GSH conjugation of xenobiotics (GSTκ1), rather than the enzymes that directly scavenge ROS (SOD1, CAT, and GPX1). SHE increases the cellular content of GSH and mitigates the oxidation of GSH to GSSG caused by PM_10_.

This study demonstrated that SHE is a useful plant extract to alleviate PM_10_-induced death of HaCaT cells. PM_10_ increased ROS production and lipid peroxidation in HaCaT cells, and these changes were moderated by SHE in a dose-dependent manner. It is suggested that SHE enhances antioxidant capacity by increasing the expression of defense genes in cells (Figure 12).

Enzymes such as SOD1, CAT, and GPX1 expressed in cells have the activity to directly remove O_2_^•−^, H_2_O_2_, and lipid peroxide [48]. GSH is a tripeptide that plays an important role in maintaining the redox balance of cells, which is used as a substrate in enzymatic reactions mediated by GPX1 and GSTκ1 and acts as a direct antioxidant [49]. GSR and G6PDH expressed in cells are involved in the regeneration of GSH [50,51], and GCL-c and GCL-m are involved in the synthesis of GSH [49,52]. The results of this study showed that SHE increased the expression of GSTκ1, GSR, G6PDH, GCL-c, and GCL-m rather than the expression of SOD1, CAT, and GPX1. This suggests that SHE selectively induces enzymes involved in the synthesis of GSH through glutamate-cysteine, the regeneration of GSH from GSSG, and GSH conjugation of xenobiotics, rather than enzymes involved in direct scavenging of ROS. Consistently, SHE increased the content of GSH and mitigated the increase in GSSG caused by PM_10_.

NRF2 is a transcription factor that induces the expression of phase II metabolism/antioxidant enzymes and plays an important function in regulating the body’s defense mechanisms [53,54]. Activation of this transcription factor is regulated by various mechanisms, and KEAP1 is one of its negative regulators [55]. Upon binding to KEAP1, NRF2 is excluded from the nucleus and degraded in the proteasome. On the other hand, when NRF2 is separated from KEAP1 by a certain stimulus, the free NRF2 enters the nucleus and can bind to the antioxidant response element (ARE) of the promoter of the target gene together with various other factors. This transactivates the expression of several genes, such as HMOX1, NQO1, GCL-c, GCL-m, and GSTκ1. In this study, SHE did not change the expression of NRF2 but decreased the expression of KEAP1. SHE increased the expression of target genes, such as HMOX1 and NQO1. This suggests that SHE activated the NRF2 system in cells.

As plant-derived substances can have positive or negative effects on cell physiology [24,25,26,27], it is important to select safe and effective types and to select their optimal concentration for use. The plant extracts tested in this study showed different or opposite effects on cell viability in the absence and presence of PM_10_. Among them, SHE was selected as a plant extract that safely and effectively alleviates the cytotoxicity of PM_10_. SHE is a mixture of various substances, and therefore the observed results are the combined effects of several components. Interesting results were obtained by comparing the effects of different solvent fractions of SHE. That is, among the solvent fractions of SHE, the BA fraction increased the cell viability whereas the MC fraction decreased the cell viability, and the other two fractions had no significant effect. The BA fraction contained a high amount of chlorogenic acid whereas the EA fraction contained a high amount of caffeic acid. Thus, we could predict that chlorogenic acid contained in the BA fraction might be an active ingredient that provided cytoprotective effects against PM_10_ toxicity. Consistently with this notion, chlorogenic acid ameliorated the death of HaCaT cells exposed to PM_10_ and reduced PM_10_-induced cellular ROS production more effectively than caffeic acid. In this study, about 100 μg mL^−1^ of SHE and 30 μM (10.6 μg·mL^−1^) of chlorogenic acid were selected as the optimal concentrations to use with high safety and cell protection efficacy.

It was previously reported that chlorogenic acid reduced oxidative stress by activating the NRF2 pathway in various in vitro and in vivo models [56,57,58]. Therefore, when combined with the results of our current study, SHE containing chlorogenic acid is suggested to mitigate PM_10_ cytotoxicity by activating the NRF2 pathway.

SHE derived from *Siegesbeckia pubescens* exhibits anti-inflammatory and wound healing properties in various in vitro and in vivo models [59,60,61]. Several terpenoids and phenolic compounds have been proposed as active ingredients that provide anti-inflammatory action [62,63,64]. Furthermore, 5,3′-dihydroxy-3,7,4′-trimethoxyflavone isolated from *Siegesbeckia pubescens* was shown to exert cytoprotective and neuroinflammatory activities in cells by involving HMOX1 induction [65]. However, there was no previous study on the cytoprotective effect of SHE derived from *Siegesbeckia pubescens* against PM toxicity. Meanwhile, an extract of tart cherry (*Prunus cerasus* L.) containing chlorogenic acid, quercetin, and kaempferol was reported to inhibit the production of ROS and the expression of apoptosis-related genes in HaCaT keratinocytes exposed to PM_10_ [66]. However, no specific experimental results were provided for chlorogenic acid itself. Therefore, our present study is the first to report the protective effect of SHE and chlorogenic acid against PM_10_.

In this study, the MTT assay was used to measure cell viability. Since particles and redox-sensitive substances can interfere with the MTT assay [67,68,69], the cells had been carefully washed with PBS to remove interfering materials before the assay. In addition, cell damage was also evaluated using the LDH release assay.

DCFH-DA was used as a fluorescent probe for measuring intracellular ROS production. Because the black pigment of PM_10_ interferes with the accurate measurement of low levels of fluorescence generated from the cells, we used a method of collecting cells from 12-well culture plates, extracting with a small volume (150 μL) of lysis buffer, centrifuging to remove the insoluble precipitate, and measuring the fluorescence of the extract, instead of directly measuring the fluorescence of the attached cells. For complementary purposes, fluorescence images of cells were additionally presented.

Immortalized HaCaT keratinocytes were used in this study and it is necessary to confirm the main findings of this study using normal human epidermal keratinocytes, 3-dimensional skin models, and in vivo animal models. It is also important to examine whether the phytochemicals have the same or different outcomes in transformed and normal cells in future studies.

## 5. Conclusions

In conclusion, the results of this study suggest that SHE derived from *Siegesbeckia pubescens* can increase the cellular antioxidant capacity through induction of defense genes, such as GCL-c, GCL-m, and G6PDH, and mitigate oxidative stress and enhance cell viability under PM_10_-exposed conditions. It is also suggested that chlorogenic acid enriched in the BA fraction of SHE may be the active phytochemical of SHE providing such antioxidant and cytoprotective effects.

## Figures and Tables

**Table 1:** Sequences of primers used for the quantitative reverse transcriptase-polymerase chain reaction (qRT-PCR) of gene transcripts.

Gene Name	GenBank Accession #	Forward (F) and Reverse (R) Primer Sequences	Reference
Catalase (CAT)	NM_001752.4	F: 5′-CATCGCCACATGAATGGATA-3′	[38]
		R: 5′-CCAACTGGGATGAGAGGGTA-3′	
Glucose 6-phosphate dehydrogenase (G6PDH)	NM_001042351.3	F: 5′-GACATCCGCAAACAGAGTGA-3′	[39]
		R: 5′-GGAGGCTGCATCATCGTACT-3′	
Glutamate-cysteine ligase-catalytic subunit (GCL-c)	NM_001197115.2	F: 5′-CTGGGAGTGATTTCTGCAT-3′	[40]
		R: 5′-AGGAGGGGGCTTAAATCTCA-3′	
Glutamate-cysteine ligase-modifier subunit (GCL-m)	NM_002061.4	F: 5′-TTTGGTCAGGGAGTTTCCAG-3′	[40]
		R: 5′-TGGTTTTACCTGTGCCCACT-3′	
Glutathione disulfide reductase (GSR)	NM_000637.5	F: 5′-CCAGCTTAGGAATAACCAGCGATGG-3′	[41]
		R: 5′-GTCTTTTTAACCTCCTTGACCTGGGAGAAC-3′	
Glutathione peroxidase (GPX) 1	NM_001329503.2	F: 5′-TTCCCGTGCAACCAGTTTG-3′	[42]
		R: 5′-GGACGTACTTGAGGGAATTCAGA-3′	
Glutathione S-transferase (GST) κ1	NM_001143679.2	F: 5′-TCTCCAGATTCCCATCCACTTCCC-3′	[43]
		R: 5′-CTGCGGCTCGGTGATGTCTTC-3′	
Glyceraldehyde 3-phosphate dehydrogenase (GAPDH)	NM_001357943.2	F: 5′-ATGGGGAAGGTGAAGGTCG-3′	[17]
		R: 5′-GGGGTCATTGATGGCAACAA-3′	
Heme oxygenase (HMOX) 1	NM_002133.3	F: 5′-CGGGCCAGCAACAAAGTG-3′	[44]
		R: 5′-ACTGTCGCCACCAGAAAGCT-3′	
Kelch-like ECH-associated protein (KEAP) 1	NM_012289.4	F: 5′-CAGAGGTGGTGGTGTTGCTTAT-3′	[45]
		R: 5′-AGCTCGTTCATGATGCCAAAG-3′	
NAD(P)H quinone oxidoreductase (NQO) 1	NM_001025434.2	F: 5′-GCACTGATCGTACTGGCTCACT-3′	This study
		R: 5′-CCACCACCTCCCATCCTTT-3′	
Nuclear factor erythroid 2-related factor (NRF) 2	NM_006164.5	F: 5′-GAGAGCCCAGTCTTCATTGC-3′	This study
		R: 5′-ACTGGTTGGGGTCTTGTGTG-3′	
Superoxide dismutase (SOD) 1	NM_000454.5	F: 5′-AGGGCATCATCAATTTCGAG-3′	[46]
		R: 5′-ACATTGCCCAAGTCTCCAAC-3′	

## Data Availability

The data presented in this study are available in this manuscript.

## Abbreviations

ARE	antioxidant response element
BA	n-butyl alcohol
CAT	catalase
DCFH-DA	2′,7′-dichlorodihydrofluorescein diacetate
DTNB	5,5-dithio-bis-2-nitrobenzoic acid
EA	ethyl acetate
G6PDH	glucose 6-phosphate dehydrogenase
GAPDH	glyceraldehyde 3-phosphate dehydrogenase
GCL-c	glutamate-cysteine ligase catalytic subunit
GCL-m	glutamate-cysteine ligase modifier subunit
GPX	glutathione peroxidase
GSH	glutathione
GSR	glutathione disulfide reductase
GSSG	glutathione disulfide
GST	glutathione S-transferase
HMOX	heme oxygenase
HPLC-DAD	high-performance liquid chromatography-photodiode array detection
IL	interleukin
KEAP	kelch-like ECH-associated protein
LDH	lactate dehydrogenase
MC	methylene chloride
MMP	matrix metalloproteinase
MTT	3-[4,5-dimethylthiazol-2-yl]-2,5-diphenyl tetrazolium bromide
NQO	NAD(P)H quinone oxidoreductase
NRF	nuclear factor erythroid 2-related factor
PBS	phosphate-buffered saline
PM	particulate matter
qRT-PCR	quantitative reverse transcriptase-polymerase chain reaction
ROS	reactive oxygen species
SHE	Siegesbeckiae Herba extract
SOD	superoxide dismutase
TBA	2-thiobarbituric acid
TBARS	2-thiobarbituric acid-reactive substance
WT	water
